# One enzyme, many reactions: structural basis for the various reactions catalyzed by naphthalene 1,2-dioxygenase

**DOI:** 10.1107/S2052252517008223

**Published:** 2017-08-08

**Authors:** Daniel J. Ferraro, Adam Okerlund, Eric Brown, S. Ramaswamy

**Affiliations:** a Department of Biochemistry, Carver College of Medicine, University of Iowa, Iowa City, IA 52242, USA; b TAS, Institute for Stem Cell Biology and Regenerative Medicine, GKVK POST, Bangalore 560 065, India

**Keywords:** naphthalene 1,2-dioxygenase, monooxygenation, sulfoxidation, deoxygenation, substrate orientation

## Abstract

Rieske nonheme iron oxygenases (ROs) are a well studied class of enzymes. Naphthalene 1,2-dioxygenase (NDO) is used as a model to study ROs. Previous work has shown how side-on binding of oxygen to the mononuclear iron provides this enzyme with the ability to catalyze stereospecific and regiospecific *cis*-dihydroxylation reactions. It has been well documented that ROs catalyze a variety of other reactions, including mono-oxygenation, desaturation, O- and N-dealkylation, sulfoxidation *etc*. NDO itself catalyzes a variety of these reactions. Structures of NDO in complex with a number of different substrates show that the orientation of the substrate in the active site controls not only the regiospecificity and stereospecificity, but also the type of reaction catalyzed. It is proposed that the mononuclear iron-activated dioxygen attacks the atoms of the substrate that are most proximal to it. The promiscuity of delivering two products (apparently by two different reactions) from the same substrate can be explained by the possible binding of the substrate in slightly different orientations aided by the observed flexibility of residues in the binding pocket.

## Abstract 2

Rieske nonheme iron oxygenases often catalyze different reactions when different substrates bind to them. From the structures of complexes of naphthalene 1,2-dioxygenase with substrates that result in different types of products, it is shown that the binding orientation of the substrate not only dictates regioselectivity and stereospecificity, but also determines the type of reaction that is catalyzed.

## 1. Introduction   

Rieske nonheme iron oxygenases catalyze a wide variety of hydroxylation reactions (Barry & Challis, 2013 ▸; Ferraro *et al.*, 2005 ▸; Parales *et al.*, 2002 ▸). They are multicomponent enzyme systems of two or three protein components consisting of electron-transport chains and an oxygenase. The electron-transport proteins transfer two electrons from NAD(P)H to the catalytic site of the oxygenase. A reductase, and often a ferredoxin, acts as an electron-transport system. The reductace contains a flavin adenine dinucleotide (FAD) and often a [2F–2S] cluster that acts as a redox center (Ensley & Gibson, 1983 ▸). The oxygenase components are either α_3_ or α_3_β_3_, where the α subunit contains a Rieske-type iron–sulfur cluster and a mononuclear iron center (Iwata *et al.*, 1996 ▸). It has been shown that molecular oxygen, a co-substrate, binds to mononuclear iron and attacks the substrate to yield products (Bugg & Ramaswamy, 2008 ▸).

Naphthalene 1,2-dioxygenase from *Pseudomonas putida* strain NCIB 9816-4 (NDO) is one of the most well studied Rieske dioxygenases; it is an α_3_β_3_ hexamer and is the first Rieske nonheme iron oxygenase for which the three-dimensional structure was determined (Kauppi *et al.*, 1998 ▸). The α subunit of NDO contains a Rieske [2Fe–2S] center that accepts electrons from the ferredoxin component, which originate from an NAD(P)H on a reductase component, and transfers them to a nonheme mononuclear iron at the active site. The catalytic domain of the terminal oxygenase is the well described 2-His/carboxylate center composed of a mononuclear iron coordinated by two histidines and one aspartic acid inside a large hydrophobic active site. NDO has a relaxed substrate specificity and catalyzes the dioxygenation of many two-ring and three-ring aromatic compounds to their respective *cis*-dihydrodiols (Hudlicky *et al.*, 1999 ▸). NDO also catalyzes a variety of other oxidation reactions, including monohydroxylation, desaturation, O- and N-dealkylation, and sulfoxidation (Resnick & Gibson, 1996 ▸; Resnick *et al.*, 1996 ▸). The extremely broad substrate range and high regioselectivities and enantioselectivities of NDO and related enzyme systems make these enzymes important not only in the degradation of environmental pollutants, but also in the biocatalytic formation of chiral synthons for the production of biologically active chemicals and pharmaceuticals (Resnick *et al.*, 1996 ▸; Boyd & Sheldrake, 1998 ▸). Recently, the enzyme RedG from *Streptomyces coelicolor* that catalyzes the oxidative carbocyclization of undecylprodigiosin to form strepto­rubin B has been shown to be a Rieske oxygenase (Sydor *et al.*, 2011 ▸). These enzymes have now also been identified in eukaryotes and play a role in cholesterol metabolism and steroid-hormone biosynthesis (Yoshiyama-Yanagawa *et al.*, 2011 ▸).

Rieske oxygenases also carry out monohydroxylation (Capyk *et al.*, 2009 ▸) and demethylation (Jiang *et al.*, 2013 ▸) reactions and these have recently been reviewed. The molecular mechanism of the dihydroxylation reaction catalyzed by NDO has been described previously (Karlsson *et al.*, 2003 ▸). It was proposed that the oxygen is activated *via* reduction by one external electron, yielding an iron(III)–(hydro)peroxo intermediate (Neese & Solomon, 1998 ▸). Mutations in this enzyme that change the regioselectivity and stereoselectivity of product formation demonstrate that this enzyme can be modified to produce products that differ from those produced by the wild type (Ferraro *et al.*, 2006 ▸). A summary of studies on NDO suggests that an activated dioxygen, bound side-on, attacks the substrate in a concerted fashion to catalyze the dihydroxylation reaction. Chakrabarty and coworkers have shown that the mechanism probably proceeds *via* a radical intermediate (Chakrabarty *et al.*, 2007 ▸).

In order to rationalize how the enzyme determines the type of reaction that is catalyzed, we have determined the structures of a series of NDO–substrate complexes. The substrates were indan, indene, styrene, ethylbenzene, phenetole, thio­anisole, ethylphenylsulfide and 1-chloronaphthalene. In order to study inhibition of the activity of NDO by inhibitory compounds, we also complexed NDO with the known inhibitors indole-3-acetate and benzamide and determined the structures of these complexes.

The studies reported in the manuscript provides a molecular basis for the hypothesis that the orientation of ligand binding not only controls the regiospecificity and stereospecificity of product formation but also the type of reaction that Rieske oxygenases catalyze on a given substrate (Kovaleva & Lipscomb, 2008 ▸).

## 2. Materials and methods   

### 2.1. Protein expression and purification   

The protein was produced using the method described in Lee *et al.* (1997 ▸). *Escherichia coli* DE3 Star protein-expression cells (Invitrogen, Carlsbad, California, USA) were transformed with the pDTG121 plasmid (Wen-Chen & Gibson, 1994 ▸). The cells were then grown in mineral salts medium (Stanier *et al.*, 1966 ▸) supplemented with 10 m*M* glucose, 150 µg ml^−1^ ampicillin and 0.6 m*M* thiamine in 1.5 l batches at 37°C until an optical density at 600 nm of 0.5–0.7 was reached. The cells were brought to 15°C and protein expression was induced with 0.4 m*M* IPTG. On induction, the cells were supplemented with 15 ml 1 *M* glucose and 0.25 m*M* ferrous ammonium sulfate. The cells were grown for 16 h with shaking at 150 rev min^−1^ at 15°C. After 16 h, the cells were harvested by centrifugation. The cell pellets were resuspended in 1 ml BTGD buffer [50 m*M* bis-tris, 10%(*v*/*v*) glycerol, 1 m*M* DTT, pH 6.8] per gram of pellet and frozen at −80°C.

The protein was purified as described previously (Lee *et al.*, 1997 ▸). The cells were lysed using a French press and then centrifuged at ∼180 000*g* for 30 min to remove cellular debris. The clarified lysate was loaded onto a 600 ml Q Sepharose FF column (GE Amersham, Piscataway, New Jersey, USA) and a 0–2 *M* potassium chloride gradient in BTGD was used for elution. Fractions containing the reddish-brown protein were pooled and concentrated using an Amicon concentrator with a YM-100 membrane. 4 *M* ammonium sulfate was added to the concentrated protein to give a final concentration of 1 *M* ammonium sulfate. The protein was loaded onto a 150 ml Octyl Sepharose FF (GE Amersham) column and eluted with a 1.0–0.0 *M* gradient of ammonium sulfate. The reddish-brown fractions were again pooled and concentrated. The concentrated protein was buffer-exchanged into 1 m*M* potassium phosphate pH 6.8. The protein was loaded onto a 40 µm Type 1 5 ml hydroxyapatite ion-exchange column (Bio-Rad, Hercules, California, USA) and eluted using a 0.001–1.0 *M* potassium phosphate gradient. The fractions containing NDO were concentrated and then buffer-exchanged into 50 m*M* MES, pH 6.8. The protein was concentrated to 60 mg ml^−1^ using Amicon Ultra centrifugal filter units with a 100 kDa molecular-weight cutoff. Protein droplets (about 1 µl drop volume) were flash-frozen in liquid nitrogen and stored at −80°C until use for crystallization.

### 2.2. Protein crystallization and complex formation   

The protein was crystallized by the hanging-drop method. 2 µl protein solution was added to 2 µl crystallization solution on a siliconized glass cover slip and placed above a well containing 0.5 ml reservoir solution. Crystals were formed in drops consisting of 1.9–2.2 *M* ammonium sulfate, 4–6% dioxane, 100 m*M* MES, pH 5.0–5.8. Drops were incubated at 6°C and crystals formed after 48–72 h. Substrates were soaked into crystals in a manner similar to that reported previously for the protein–naphthalene complex (Karlsson *et al.*, 2003 ▸). 1 µl ethanol saturated with substrate was added to 19 µl crystallization solution and the crystals were transferred to the soaking solution for at least two weeks to allow the substrate to bind.

### 2.3. Data collection, processing, structure solution and refinement   

X-ray diffraction data from the wild-type NDO complexes were collected on the IMCA-CAT beamline 17-ID at the Advanced Photon Source, Argonne National Laboratory using an ADSC Quantum 210 CCD detector or on the MBC beamline 4.2.2 at the Advanced Light Source at Lawrence Berkeley National Laboratory using a NOIR-1 CCD detector. Data-collection and processing statistics are reported in Table 1 ▸. The data were processed using d*TREK (Pflugrath, 1999 ▸). A previous structure of wild-type NDO (PDB entry 1o7w; Karlsson *et al.*, 2003 ▸) was used directly as a starting model for refinement. Refinement was initially performed using *REFMAC*5 (Murshudov *et al.*, 2011 ▸) in the *CCP*4 software suite v.6.0.0 (Winn *et al.*, 2011 ▸). The molecular-visualization program *Coot* (Emsley & Cowtan, 2004 ▸) was used for model building and visualization. The ligands were modeled into the electron density and their position was refined along with the protein in the *PHENIX* software suite (Adams *et al.*, 2011 ▸). Solvent-molecule positions were verified using a composite OMIT map with the *SOLVENT-OMIT* software (Brown, 2008 ▸), and new solvent molecules were modeled where electron density was present. Images were rendered and r.m.s.d. calculations were performed using *PyMOL* (DeLano, 2000 ▸).

## 3. Results   

### 3.1. Complexes with indene (PDB entry 4hm5) and indan (PDB entry 4hm4)   

**Figure 1:**
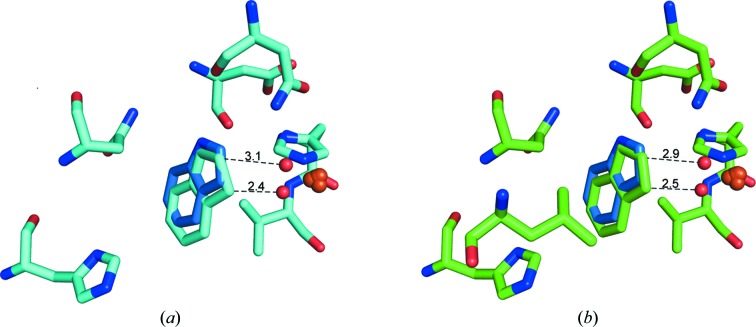
Wild-type NDO in complex with indan (magenta) (*a*) and with indene (green) (*b*) overlapped with that with indole (dark blue). Active-site residues of NDO are represented by sticks with green and cyan C atoms. The position of indole, from PDB entry 1o7n, is represented by blue C atoms. The mononuclear iron (overlapping orange spheres show the Fe position in the different structures reported here to illustrate the small movements in the Fe position) and modeled dioxygen (from PDB entry 1o7n; small red spheres) are also shown.

NDO catalyzes a monohydroxylation reaction when indene or indan are the substrate. Indan reacts to form (*S*)-1-indanol/(*R*)-1-indanol. The monohydroxylation of indene results in the formation of (*S*)-1-indenol/(*R*)-1-indenol (Gibson *et al.*, 1995 ▸; Wackett *et al.*, 1988 ▸). Indan also undergoes a desaturation reaction to form indene. Indene also forms dihydroxylated products, with (1*R*,2*S*)-indenediol as the major isomer; trace amounts of other isomers are also produced. The crystal structures of indan and indene in complex with NDO were refined to 1.6 and 1.5 Å resolution, respectively, and the resulting statistics are summarized in Table 1 ▸. The orientation of the substrate in the active site is similar to that of indole (Fig. 1 ▸). Evaluation of the electron density of substrate binding only shows the possibility of the five-membered ring being placed towards the iron center. The six-membered ring is bound between Val209 and Leu307, residues that are believed to anchor the second ring of substrates in the active site. The position of indole in PDB entry 1o7n is represented by blue C atoms. The mononuclear iron and modeled dioxygen based on the ternary complex with indole and dioxygen are also shown. However, in the structures reported here there is no bound dioxygen, only a water molecule bound to the mononuclear iron. The C1 and C2 atoms of the bound substrate are closest to the modeled dioxygen. Biotransformation experiments also show that all of the products formed are formed by attack of the radical oxygen on the C1 and C2 atoms.

### 3.2. Complexes with styrene (PDB entry 4hm7) and ethylbenzene (PDB entry 4hm3)   

**Figure 2:**
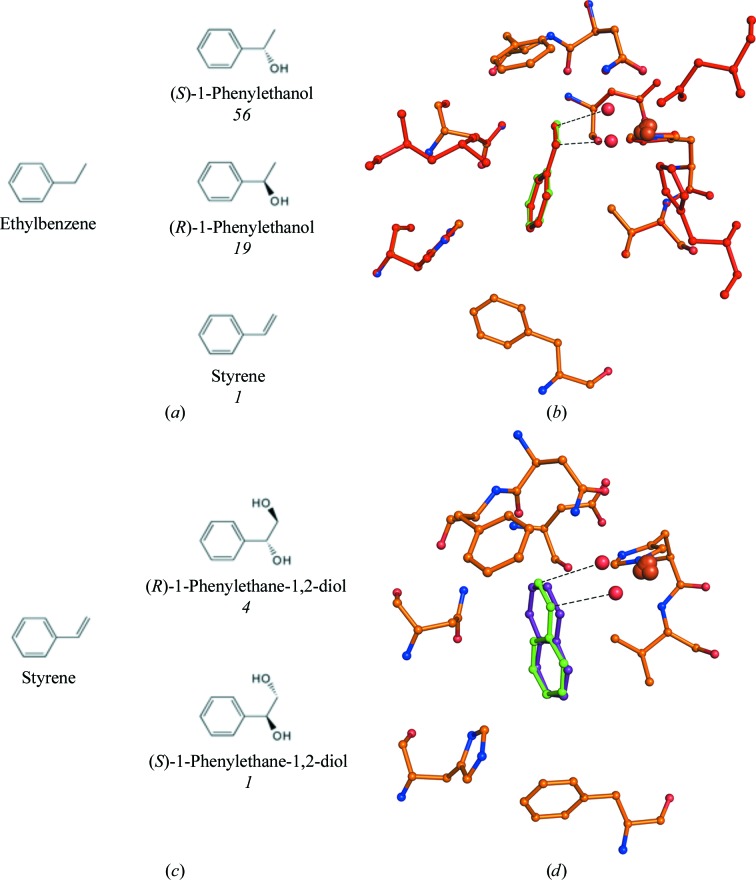
(*a*, *c*) Products formed with ethylbenzene (*a*) and styrene (*c*) as substrates. Approximate ratios of product detected in biotransformation assays are shown in italics below the products. (*b*, *d*) Wild-type NDO in complex with styrene (green) overlapped with that with naphthalene (purple) (*d*) and that with ethylbenzene (red) overlapped with that with styrene (*b*). Active-site residues are represented by sticks with orange C atoms. The mononuclear iron (large orange sphere) and modeled dioxygen from PDB entry 1o7n (small red spheres) are also shown.

NDO catalyzes a vinyl-group dihydroxylation reaction with styrene to produce 1-phenyl-1,2-ethanediol, in which formation of the *R* isomer is favored over the *S* isomer in an approximately 3:1 ratio (Lee & Gibson, 1996*a* ▸). Ethylbenzene undergoes monohydroxylation of the benzylic C atom to form 1-phenethyl alcohol and desaturation to form styrene (Fig. 2 ▸; Lee & Gibson, 1996*b* ▸). The structures of NDO in complex with styrene and ethylbenzene were both determined to 1.6 Å resolution. Crystallographic data-collection and refinement statistics are reported in Table 1 ▸. For both ethylbenzene (Figs. 2 ▸ *a* and 2 ▸ *b*) and styrene (Figs. 2 ▸ *c* and 2 ▸ *d*), the ligands were found in the active site in multiple orientations based on the electron density. One of the orientations that can be modeled would include the aromatic ring at a position similar to that of the hydroxylated C atoms of naphthalene and suggests that a dihydroxylated product would be formed (Supplementary Figs. S1*a* and S1*b*). However, the dihydroxylated product was not observed in biotransformation assays.

### 3.3. Complexes with phenetole (PDB entry 4hm6), thioanisole (PDB entry 4hm8) and ethylphenylsulfide (PDB entry 4hm2)   

**Figure 3:**
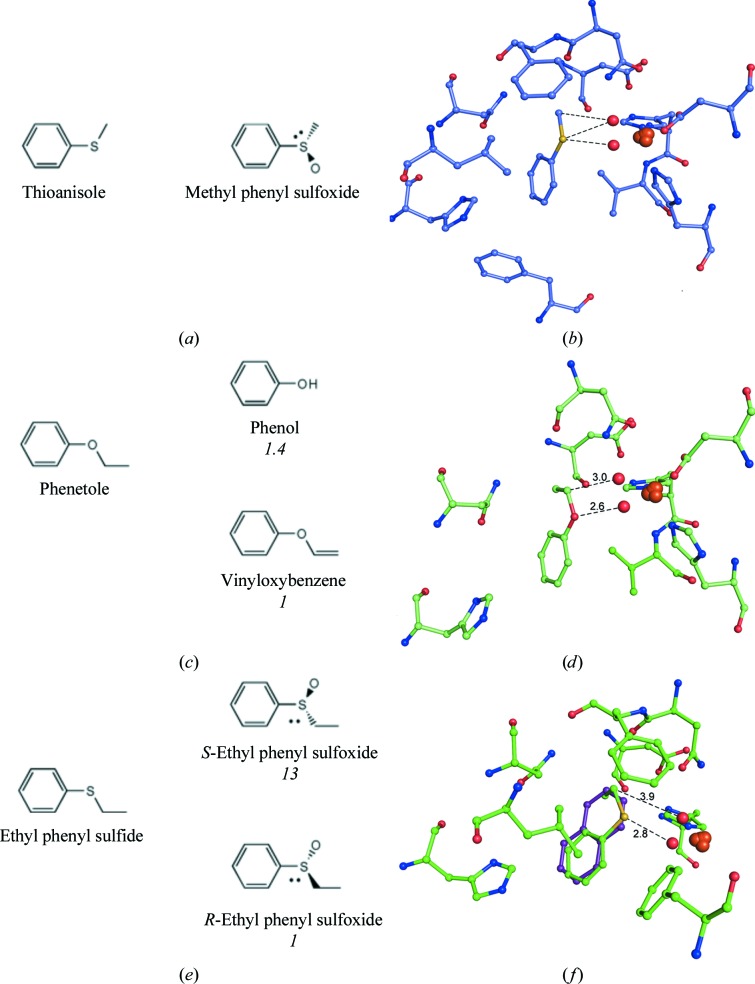
Products formed by NDO with thioanisole (*a*), phenetole (*c*) and ethylphenylsulfide (*e*). Approximate ratios of products detected in biotransformation assays are shown in italics below the products. Wild-type NDO in complex with phenetole (green) (*d*), with ethylphenylsulfide (green) overlapped with that with naphthalene (purple) (*f*) and with thioanisole (green) (*b*), with active-site residues of NDO represented by sticks with green C atoms. The mononuclear iron (large orange sphere) and modeled dioxygen from PDB entry 1o7n (small red spheres) are also shown.

Phenetole was chosen as a representative compound for structural analysis of the dealkylation reaction. It forms phenol on dealkylation. However, on desaturation of the ethyl group it forms vinyloxybenzene (Fig. 3 ▸; Resnick & Gibson, 1993 ▸). Similarly, thioanisole and ethylphenylsulfide were chosen as representative compounds that undergo sulfoxidation for complex formation and subsequent crystallographic analysis. NDO catalyzes the sulfoxidation of both of these compounds to form the respective sulfoxides (Fig. 3 ▸; Kerridge *et al.*, 1999 ▸; Lee *et al.*, 1995 ▸). The structures of phenetole, thioanisole and ethylphenylsulfide in complex with NDO were solved to 1.6, 1.4 and 1.5 Å resolution, respectively, and crystallo­graphic details are summarized in Table 1 ▸. Fig. 3 ▸(*b*) shows thioanisole bound in the active site of NDO. A 1.0σ (2*F*_o_ − *F*_c_) electron-density map shows two positions of the S atom arising from two possible orientations of binding (Supplementary Fig. S1*c*). One position places the sulfur near the mononuclear iron at a distance of ∼4.3 Å, which is close enough for oxidation by the enzyme (Fig. 3 ▸ *b*). In the ethylphenylsulfide complex the S and C atoms (S—CH_2_) are 2.8 and 3.9 Å away from the molecular oxygen, respectively (Fig. 3 ▸ *f*), while in the phenetole complex the O and C atoms (O—CH_2_) are 2.6 and 3.0 Å away from the molecular oxygen, respectively (Fig. 3 ▸ *d*), which suggests that desaturation and de­alkylation reactions would not be possible for ethylphenylsulfide, and this was not observed in the biotransformation assay.

### 3.4. Complex with 1-chloro­naphthalene (PDB entry 4hjl)   

**Figure 4:**
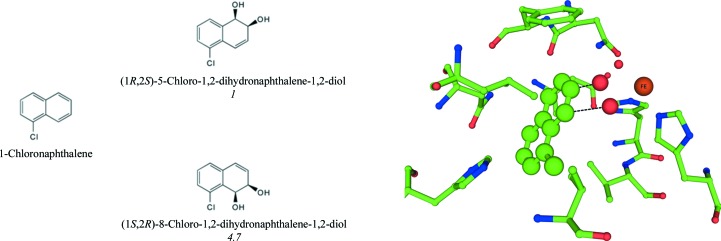
Products formed by NDO with chloronaphthalene. Approximate ratios of products detected in biotransformation assays are shown in italics below the products. Wild-type NDO in complex with chloronaphthalene is shown in green. Active-site residues of NDO are represented by sticks with green C atoms. The mononuclear iron (large orange sphere) and modeled dioxygen from PDB entry 1o7n (small red spheres) are also shown.

In the case of 1-chloronaphthalene, NDO dihydroxylates the nonhalogenated ring. The C atoms on the same side of the molecule as the Cl atom are in the closest proximity to the modeled dioxygen. This results in the formation of (1*R*,2*S*)-8-chloro-1,2-dihydro­naphthalene-1,2-diol as the major product and (1*R*,2*S*)-5-chloro-1,2-dihydronaphthalene-1,2-diol as the minor product (Fig. 4 ▸). The structure of NDO in complex with 1-choloronaphthalene was determined to 1.5 Å resolution. Crystallographic data-collection and refinement statistics are reported in Table 1 ▸. 1-Chloronaphthalene was found to bind in a position similar to naphthalene, interacting with Val209 and Leu307 above and below the substrate (Fig. 4 ▸). The Cl atom was found to sit near the entrance of the active site in an area that is relatively open.

### 3.5. Complexes with benzamide (PDB entry 4hkv) and indole-3-acetate (PDB entry 4hm0)   

**Figure 5:**
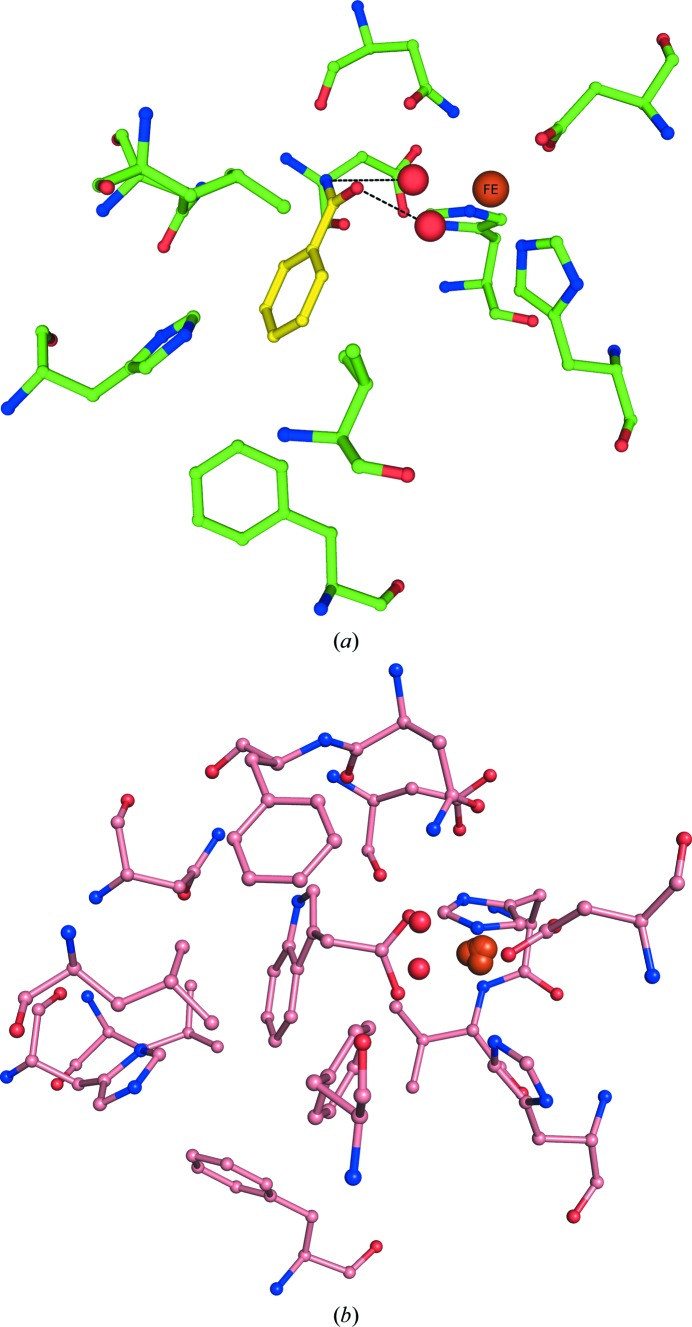
(*a*) Active-site residues of NDO with benzamide (yellow) bound are represented by sticks with green C atoms. The mononuclear iron (large orange sphere) and modeled dioxygen from PDB entry 1o7n (small red spheres) are also shown. (*b*) Active-site residues of NDO bound to indole-3-acetate. The mononuclear iron (large orange sphere) and modeled dioxygen from PDB entry 1o7n (small red spheres) are also shown.

The structure of NDO in complex with benzamide was determined to 1.65 Å resolution. Crystallographic data-collection and refinement statistics are reported in Table 1 ▸. The inhibitor binds in the active site in one orientation (Fig. 5 ▸ *a*). The N atom of the substrate forms a hydrogen bond to the water/hydroxide that is bound to the mononuclear iron. The O atom of the inhibitor interacts with the side-chain N atom of Asn201, which is 3.6 Å away, and the side-chain N atom of Asn297, which is 4.0 Å away. This also positions the aromatic ring between Val209 and Leu307, residues that have been shown to anchor the second ring of aromatics in the active site of NDO and other similar ROs (Fig. 5 ▸ *a*).

The structure of NDO in complex with indole-3-acetate was determined to 1.8 Å resolution. Crystallographic data-collection and refinement statistics are reported in Table 1 ▸. The inhibitor (a product analog) binds in the active site in one orientation, based on the electron density (Fig. 5 ▸ *b*). Both O atoms of the carboxylate group of the inhibitor coordinate the catalytic metal, with distances of 2.3 and 2.1 Å from the mononuclear iron. For consistency, Fig. 5 ▸(*b*) also shows the modeled dioxygen. However, it is clear that the carboxylate atoms occupy positions that are very close to the O atoms of the dioxygen in the substrate complex. Asn201 also forms a hydrogen bond to one of the carboxylate O atoms, stabilizing the orientation. The six-membered aromatic ring of the substrate is also between Val209 and Leu307, a region of the active site that is believed to stabilize the placement of the aromatic ring of the substrate. The N atom of indole-3-acetate interacts with the backbone O atom of Asp205 and the side chain of Asn297, further stabilizing the orientation (Fig. 5 ▸ *b*).

## 4. Discussion   

In this study, we have reported structures of NDO in complex with ten different ligands. These ligands (substrates and inhibitors) belong to different classes: eight are substrates and two are inhibitors. Many indan-like compounds, such as indene, indole and indanol, have been determined to be substrates of NDO. Dihydroxylation, monohydroxylation and desaturation reactions have all been shown to be catalyzed by NDO on this set of compounds (Gibson *et al.*, 1995 ▸). The structure reported here shows that indole is anchored in the active site by interactions between the substrate N atom and the active-site wall. In the case of indene, monohydroxylated and dihydroxylated product forms were detected in the biotransformation assays. Naphthalene only forms a dihydroxylated product, while indene and indan form different products. While not visible in our crystal structures, the extra wriggle space available allows the substrate to undergo minor changes in orientation. It is possible that these minor changes in orientation will result in the formation of monohydroxylated or dihydroxylated products. As in previous reports, this work also suggests that the regioselectivity and stereoselectivity of product formation is controlled by orientation. Similarly, with indan, the choice of either monooxygenation or desaturation is probably a combination of both orientation and energetics. The same C atoms are positioned near the mononuclear iron in both cases. The ratio of products formed is most likely to be determined by the energetics of the reaction.

The structures of NDO complexed with styrene, ethyl­benzene, thioanisole, phenetole and ethylphenylsulfide also suggest that orientation alone controls the product regio­selectivity and stereoselectivity. All of these structures show the substrate modeled in multiple positions. In every case at least one of the positions modeled suggest a product to be formed, usually a ring dihydrodiol, that is not observed in biotransformation assays. In these instances the potential atoms vary in reactivity, and in virtually all of the cases the product formed is a result of the more reactive atom(s) being oxidized. It is also possible that the presence of an aromatic ring is essential at the farther site for dioxygen activation. This theory is supported by the studies of Lee, where the addition of benzene to the protein results in oxygen activation followed by radical formation, but a lack of any product formation: a phenomenon the authors described as an uncoupling of the electron transport from the binding of the substrate close to the mononuclear iron (Lee, 1999 ▸).

Benzamide does bind in one orientation in the active site of NDO. It was hypothesized that this compound should bind tightly with the heteroatoms near the mononuclear iron because the aromatic ring could be positioned between Val209 and Leu307. In addition, the amide group would be able to interact with charged residues on the wall of the active site, while forming hydrogen bonds to the water/hydroxide bound to the mononuclear iron. X-ray structures confirm this hypothesis. There are no examples of NDO catalyzing the oxidation of compounds containing an amide group and it is unlikely, based on structural evidence, that NDO would be able to catalyze the oxidation of substrates with amide groups that are free to interact with the mononuclear iron very efficiently. In contrast, NBDO-O_JS765_ would likely be able to oxidize these substrates (Lessner *et al.*, 2002 ▸; Friemann *et al.*, 2003 ▸).

Structures of NDO in complex with product have shown that the mononuclear iron is capable of binding directly to a dihydroxylated substrate through interactions with both hydroxyl groups (Karlsson *et al.*, 2003 ▸). Based on this information, we hypothesized that the mononuclear iron should coordinate carboxylate groups directly, thus controlling the substrate orientation. The structure with indole-3-acetate shows this to be the case. No biotransformation data are available for this compound; however, a similar compound, 2-napthoic acid, is a substrate of NDO (Brilon *et al.*, 1981 ▸). This is the only carboxylate-containing compound reported to be have been tested as a substrate, and it is unique with respect to the regioselectivity of product formation: oxidizing the ring carbon with the carboxylate substituent along with the adjacent ring carbon.

**Figure 6:**
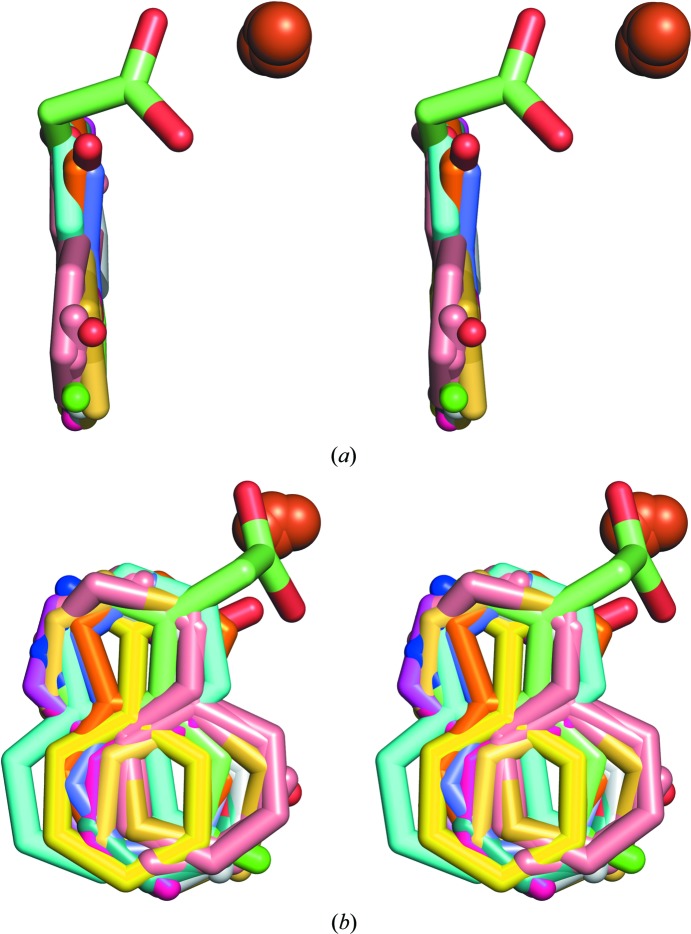
Stereo diagram of a superposition of all of the ligands discussed in the manuscript from two different views: (*a*) perpendicular to the plane of the aromatic rings and (*b*) parallel to the plane of the aromatic rings.

**Figure 7:**
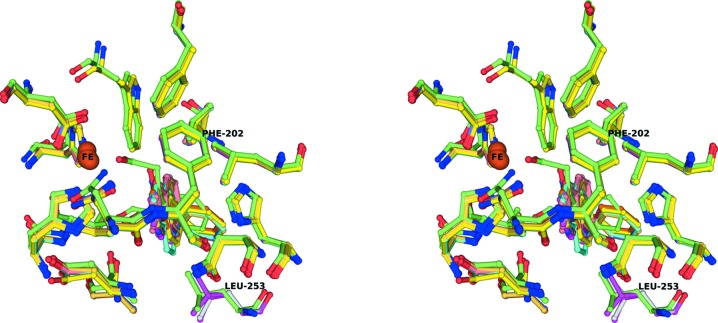
Stereo diagram of a superposition (from all structures discussed in this manuscript) of the residues in the active site, illustrating the changes in conformation of the residues that bind the mononuclear iron and the substrate. The Fe atom, Phe202 and Leu253 are labeled.

In the case of biphenyl dioxygenase, docking studies have shown that ligands can bind in multiple conformations and also that the ligand binds first to form a product and binds again to undergo a second reaction (Pham & Sylvestre, 2013 ▸; Pham *et al.*, 2012 ▸). It is quite possible that in the case of NDO the product of one reaction binding again as a substrate does occur. The multiple ratios of the different products formed from the same substrate suggests that in the large pocket of NDO multiple cycles of reaction can take place when smaller substrates are involved: for example, indan could first be converted to indene and then to a monohydroxylated or dihydroxylated product of indene. However, given the ability for multiple modes of binding, different products can also be formed. This supports the idea proposed by Kovelva and Lipscomb that substrate binding initiates the formation of the hydroperoxide, which then attacks the substrate (Kovaleva & Lipscomb, 2008 ▸). Interestingly, on superposition of all of the different complexes onto each other and looking at the possible size of the pocket (Fig. 6 ▸), it is clear that in all cases the pocket is very flat and most of the ligands bind with very little deviation. The conformations that are shown in Fig. 6 ▸ are from previously published work as well as from this study. While some substrates can be modeled in multiple orientations in our study (Supplementary Fig. S1), only orientations that may result in products that have been verified in bio­transformation studies are presented. We contend that the other orientations are unproductive binding and hence are functionally not relevant. More interestingly, a superposition of all of the different structures to understand the possible flexibility of the amino acids in the substrate-binding pocket also reveals that Leu253 and Phe224 show the maximum flexibility (Fig. 7 ▸). This confirms the idea that by changing a few residues in the binding pocket one can control the selectivity of the substrates that can bind as well as the orientation, as has been shown by us for NDO and as has also been shown for carbazole dioxygenase (Inoue *et al.*, 2014 ▸; Nojiri *et al.*, 1999 ▸). Recent work has also shown that it is also possible to achieve selectivity by controlling entry to the active site (Escalante *et al.*, 2017 ▸).

It is tempting, based on these structures and other structures of Rieske oxygenases which catalyze oxygen-dependent reactions on a variety of substrates, to propose that the primary catalytic role of the enzyme is the production of the oxygen radical when substrate binds, if molecular oxygen and electrons are available. The residues that anchor the Rieske cluster and those that hold the mononuclear iron center (the 2-His/carboxylate triad) and the connection between the two centers are conserved, while the residues that hold the substrate in the active site are not conserved and dictate the type of substrate (selectivity) and the orientation of the substrate binding to the active site. These residues then play a role in the exact nature of orientation of the substrate towards the produced oxygen-radical species and thereby determine product formation. Given that many of these enzymes have evolved to activate inert environmental pollutants for use as carbon sources, this evolutionary strategy optimizes the preservation of core function (the generation of a high-energy radical species) and yet allows adaptation to a variety of carbon sources that may need to be degraded.

## Supplementary Material

PDB reference: NDO, complex with 1-chloronaphthalene, 4hjl

PDB reference: complex with benzamide, 4hkv

PDB reference: complex with indole-3-acetate, 4hm0

PDB reference: complex with ethylphenylsulfide, 4hm2

PDB reference: complex with ethylbenzene, 4hm3

PDB reference: complex with indan, 4hm4

PDB reference: complex with indene, 4hm5

PDB reference: complex with phenetole, 4hm6

PDB reference: complex with styrene, 4hm7

PDB reference: complex with thioanisole, 4hm8

Supplementary Figure S1.. DOI: 10.1107/S2052252517008223/mf5018sup1.pdf

## Figures and Tables

**Table:** Values in parentheses are for the highest resolution shell.

Ligand bound (PDB code)	1-Chloronaphthelene (4hjl)	Benzamide (4hkv)	Ethylbenzene (4hm3)	Ethylphenylsulfide (4hm2)	Indan (4hm4)
Resolution range (Å)	44.7–1.5 (1.55–1.50)	47.9–1.6 (1.71–1.65)	15.9–1.5 (1.55–1.50)	47.9–1.6 (1.72–1.60)	15.9–1.5 (1.55–1.50)
Unit-cell parameters (Å)					
*a* = *b*	139.9	140.1	139.9	140.0	140.1
*c*	208.4	208.5	208.3	208.3	208.2
Total reflections	883187	898724	490726	366476	836020
Unique reflections	124386 (12332)	94230 (9183)	124136 (11929)	101718 (19301)	124179 (11784)
Multiplicity	7.1 (6.8)	9.5 (9.1)	3.9 (2.5)	3.6 (3.4)	6.7 (4.3)
Completeness (%)	99.47 (99.48)	100 (100)	99.52 (96.57)	97.3 (93.2)	99.4 (95.1)
Mean *I*/σ(*I*)	11.9 (3.7)	9.4 (2.5)	7.3 (1.8)	12.3 (4.4)	9.6 (2.5)
Wilson *B* factor (Å^2^)	13.5	17.74	13.23	12.17	13.9
*R*_merge_	0.066 (0.368)	0.104 (0.537)	0.097 (0.517)	0.064 (0.503)	0.101 (0.584)
*R* factor	0.145 (0.199)	0.165 (0.231)	0.165 (0.328)	0.167 (0.426)	0.158 (0.285)
*R*_free_	0.177 (0.262)	0.202 (0.259)	0.203 (0.347)	0.206 (0.385)	0.196 (0.326)
No. of atoms					
Total	5995	6197	6067	6091	5848
Macromolecules	5083	5083	5093	5083	5083
Ligands	68	66	73	93	66
Water	844	1048	901	915	699
Protein residues	638	638	638	653	653
R.m.s.d., bond lengths (Å)	0.006	0.007	0.007	0.007	0.006
R.m.s.d., angles (°)	1.19	1.2	1.15	1.14	1.16
Ramachandran favored (%)	97	97	97	97	97
Ramachandran outliers (%)	0	0.16	0.16	0.16	0.32
Average *B* factor (Å^2^)	18.3	23.1	17.7	18.5	18.6

**Table:** 

Ligand bound (PDB code)	Indene (4hm5)	Phenetole (4hm6)	Styrene (4hm7)	Thioanisole (4hm8)	Indole-3-acetate (4hm0)
Resolution range (Å)	16–1.5 (1.55–1.50)	15.9–1.5 (1.55–1.50)	15.9–1.5 (1.55–1.50)	15.0–1.3 (1.35–1.30)	40.6–1.8 (1.86–1.80)
Unit-cell parameters (Å)					
*a* = *b*	139.9	139.8	139.8	140.2	140.7
*c*	208.2	208.2	208.0	208.3	207.8
Total reflections	626864	931709	494527	761227	333830
Unique reflections	110048 (11326)	122798 (10773)	121577 (10390)	190595 (18186)	66787 (6627)
Multiplicity	5.7 (3.4)	7.6 (5.1)	4.1 (2.9)	4.0 (2.5)	5.0 (4.7)
Completeness (%)	88.2 (91.7)	98.42 (87.22)	97.77 (84.38)	99.4 (95.6)	91.2 (91.5)
Mean *I*/σ(*I*)	10.5 (2.1)	9.9 (2.6)	11.4 (2.3)	8.1 (1.6)	6.1 (2.1)
Wilson *B* factor (Å^2^)	15.97	15.67	15.21	11.69	26.65
*R*_merge_	0.068 (0.516)	0.088 (0.591)	0.057 (0.436)	0.083 (0.529)	0.137 (0.493)
*R* factor	0.160 (0.286)	0.151 (0.280)	0.157 (0.288)	0.167 (0.348)	0.207 (0.270)
*R*_free_	0.204 (0.321)	0.187 (0.318)	0.193 (0.319)	0.193 (0.348)	0.247 (0.302)
No. of atoms					
Total	5757	5966	5812	6109	5655
Macromolecules	5083	5083	5083	5073	5089
Ligands	66	75	73	65	103
Water	608	808	656	971	463
Protein residues	653	641	653	644	651
R.m.s.d., bond lengths (Å)	0.006	0.007	0.007	0.006	0.008
R.m.s.d., angles (°)	1.21	1.16	1.16	1.17	1.23
Ramachandran favored (%)	97	97	97	97	98
Ramachandran outliers (%)	0.16	0.16	0.16	0.2	0.16
Average *B* factor (Å^2^)	21.3	20.9	20.2	16.8	29.4
