# Supplementary material for: One enzyme, many reactions: structural basis for the various reactions catalyzed by naphthalene 1,2-dioxygenase
Source: IUCrJ. 2017 Aug 8;4(Pt 5):648–56. doi: 10.1107/S2052252517008223 (PMC5619856; doi:10.1107/S2052252517008223)
Supplement: Supplementary file 1 [file m-04-00648-sup1.pdf]

IUCrJ

Volume 4 (2017)

Supporting information for article:

One enzyme many reactions: structural basis for the various reactions catalyzed by naphthalene 1,2-dioxygenase

Daniel Ferraro, Adam Okerlund, Eric Brown and S. Ramaswamy

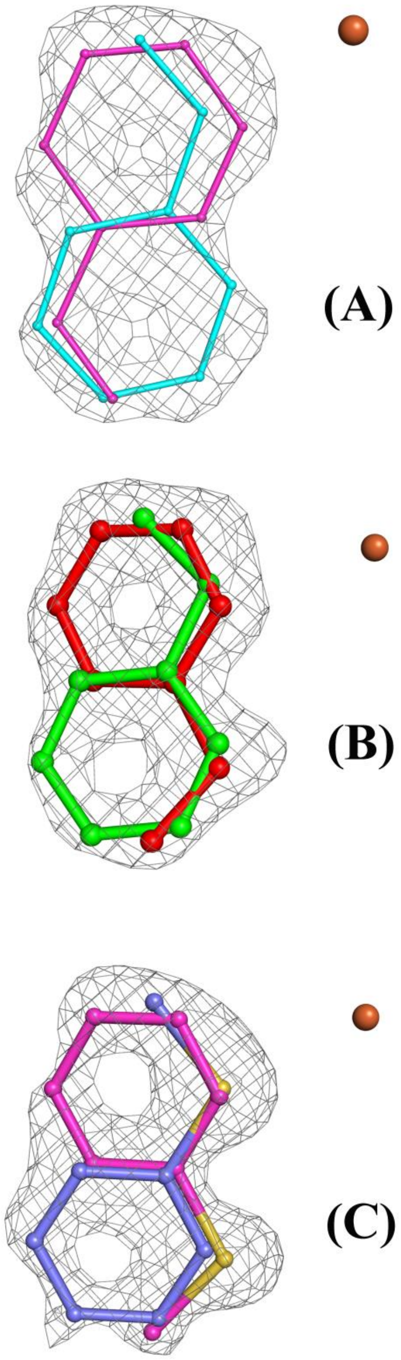

Figure S1 For all the three figures (A), (B) and (C), the maps are the final refined (2Fo-Fc) maps contoured at one sigma. The two different orientations in which the ligands could be modeled in the electron density maps is shown. (A) styrene, (B) ethylbenzene and (C) thioanisole. Refinement was carried out with both conformations present in equal occupancy. In each of these cases the biotransformation studies do not show any product where there is a modification in the aromatic ring. While, the electron density maps allow us to model the ligand in multiple ways, we believe the only productive orientation is the one where the ring is further from the mono-nuclear iron (shown as orange ball). In the main text, hence only the functionally relevant orientations are discussed.
